# Central Nervous System Delivery of Antibodies and Their Single-Domain Antibodies and Variable Fragment Derivatives with Focus on Intranasal Nose to Brain Administration

**DOI:** 10.3390/antib10040047

**Published:** 2021-11-30

**Authors:** Arghavan Soleimanizadeh, Heiko Dinter, Katharina Schindowski

**Affiliations:** 1 Institute of Applied Biotechnology, Biberach University of Applied Science, 88400 Biberach, Germany; soleimani@hochschule-bc.de (A.S.); Heiko.Dinter@web.de (H.D.); 2 Faculty of Medicine, University of Ulm, 89081 Ulm, Germany; 3 Department of Pharmacy and Biochemistry, University of Tübingen, 72076 Tübingen, Germany

**Keywords:** intranasal delivery, scaffolds, VHH, VNAR, Fc receptor, mucosal transport

## Abstract

IgG antibodies are some of the most important biopharmaceutical molecules with a high market volume. In spite of the fact that clinical therapies with antibodies are broadly utilized in oncology, immunology and hematology, their delivery strategies and biodistribution need improvement, their limitations being due to their size and poor ability to penetrate into tissues. In view of their small size, there is a rising interest in derivatives, such as single-domain antibodies and single-chain variable fragments, for clinical diagnostic but also therapeutic applications. Smaller antibody formats combine several benefits for clinical applications and can be manufactured at reduced production costs compared with full-length IgGs. Moreover, such formats have a relevant potential for targeted drug delivery that directs drug cargo to a specific tissue or across the blood–brain barrier. In this review, we give an overview of the challenges for antibody drug delivery in general and focus on intranasal delivery to the central nervous system with antibody formats of different sizes.

## 1. General Overview on Immunoglobulin Structures

Antibodies, otherwise known as immunoglobulins (Igs), are glycoprotein molecules produced by plasma cells and are mostly found in blood and lymphoid tissues. The primary function of antibodies in vivo is to recognize and neutralize infectious agents, such as pathogenic bacteria and viruses. Antibodies are directed against various antigens and play a pivotal role in the defense mechanism of higher vertebrates and are also involved in autoimmune diseases and allergies. They are well-characterized molecules because of their considerable use in research, diagnostics and therapy [1]. Antibody-based therapy, with currently more than 100 approved monoclonal-derived products, has emerged as a class of novel therapeutics for various diseases [2]. This type of therapy has grown to become the dominant product class within the biopharmaceutical market and their market is still rapidly growing. Thanks to antibody engineering techniques, it is now possible to generate a specific antibody against almost any protein or peptide antigen [3,4]. Hence, antibodies can be used as therapy for many human disorders, including central nervous system (CNS) diseases, such as Parkinson’s disease [5], multiple sclerosis [6], amyotrophic lateral sclerosis [7] and Alzheimer’s dementia [8,9].

**Figure 1:**
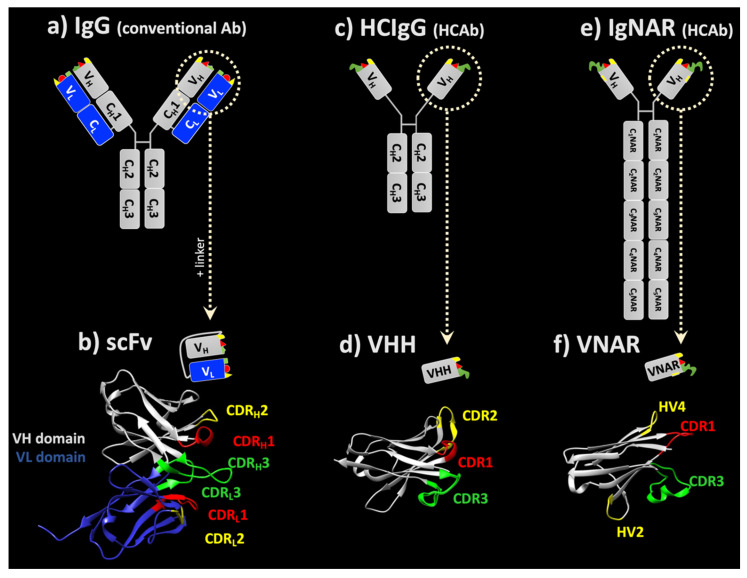
Schematic representation of scaffolds derived from human IgG or heavy-chain-only antibodies (HCAbs). The variable fragment (Fv) of a conventional IgG (**a**) can be fused with a polypeptide linker to form an scFv (**b**). Isolated VH or VL domains can also be expressed as human-derived sdAbs. The isolated VH of a (**c**) camelid heavy chain IgG (HCIgG) is termed VHH (**d**) and has a more compact structure compared to a VH derived from conventional IgG. Although the shark immunoglobulin new antigen receptor (IgNAR) is the largest antibody (**e**), its isolated VH domain, VNAR (**f**), is the smallest sdAb and provides the most compact structure. Crystal structures of a representative (**b**) scFv (6S2I; [93]), (**d**) lama VHH domain (1I3V; [94]) and (**f**) VNAR (1SQ2; [85]) were retrieved from PBD. CDR regions were indicated with Kabat numbering using AbRSA tools [95]; indices in the scFv refer to HC and LC, respectively. The structures were visualized using UCSF Chimera software.

Igs are Y-shaped molecules with a distinct proteolytic fragmenting pattern: after an IgG antibody is digested with the papain protease, for instance, it is cleaved into two distinct fragments: (i) a fragment antigen binding (Fab) with around 45 kDa and (ii) the crystallizable fragment (Fc) with approximately 50 kDa [10]. IgG antibodies consist of two identical light chains (LC) and two identical heavy chains (HC). Each HC and LC consists of two regions (Figure 1a) [11,12]: the variable (V) region and the constant (C) region which are located at the N-terminus and the C-terminus of the antibody molecule, respectively [13]. In humans, the HC consists of a variable (VH) domain and up to four constant domains (CH). The HCs are stabilized by varying numbers of disulfide bonds at the so-called hinge region. The hinge region is located between the first and second constant domain of heavy chains (CH) and is responsible for the flexibility of the two fragment-antigen-binding arms of antibodies. 

Each LC has one variable domain (VL) and one constant domain (CL). Two types of LC are encoded by different genes on two distinct chromosomes: kappa (κ) and lambda (λ). Each antibody naturally includes only one of these LC types. In humans, the average κ:λ ratio is 2:1, but the ratio is different in other species. The explanation for this dissimilitude is not known. However, this ratio can be used to detect unusual B-cell proliferation by observing a shift in the κ:λ ratio [14]. 

The variable domains of antibodies have an immunoglobulin fold, designating a protein domain structure, first discovered in immunoglobulin constant and variable domains, which consists of two β-sheets packed against each other [15]. The binding of these two domain sheets is facilitated via several disulfide bonds and hydrophobic interactions [16]. This constitution is essential for the function of antibodies through the formation of potential binding sites at the loops at the end of the structure. These hypervariable loops are called complementary determining regions (CDRs), which consist of hypervariable regions and appear in antibodies and T cell receptors. The amino acid sequence in these loops is highly variable, which leads to the production of several antibodies that have the ability to bind to a variety of antigens [16]. Each variable region in the HC and LC domains has three CDRs between four framework (FR) sequences. The FR region with a highly conserved sequence plays an essential role in the conservation and stability of the structure of the domain antibody [17,18]. 

Most paratopic regions of an antibody molecule are located in the CDRs of VH and VL domains. Paratopic regions, or antigen-binding sites, refer to the parts of an antibody that recognize an antigen and bind to it. Each antigen binds to the antibody noncovalently through highly specific interactions. The affinity of the antibody refers to the strength of the reaction of a single epitope to a paratope. There are two identical antigen-binding sites on each full-length antibody molecule; thus, they can interact with two equivalent antigens at the same time. This increases the binding strength of the antigen–antibody complex. The resulting accumulated strength of multiple affinities is called avidity [19]. CDR3 is the most extended loop out of the other CDR loops and plays a paramount role in antigen binding [18]. The length of CDR3 exhibits vast diversity in different species [20,21]. 

Isolated VH or VL, or similar isolated single variable domains, are called single-domain antibodies (sdAbs). These small antibody-derived scaffolds can be screened and produced with simple biotechnological tools. Camelids and sharks express so-called heavy-chain-only antibodies that are specifically appropriate to use as blueprints for sdAbs (see details below). Some sdAbs have been approved as therapeutics, but still their number in clinical trials is low compared to full-length IgGs. Here we discuss their potential but also their challenges in drug delivery.

## 2. General Considerations Relating to the Distribution and Tissue Penetration of Full-Length IgG Antibodies 

Using antibodies has revolutionized targeted therapy because of their specificity and unique properties, drawing the attention of several major pharmaceutical companies to allocate part of their research to the development and production of therapeutic IgGs and antibody–drug conjugates (ADCs) [22]. Despite the enormous success of monoclonal antibodies (mAbs), in particular, as drugs for treating cancer and immune diseases, they have numerous disadvantages because of their size and complex structure. Apart from their limited biodistribution, the production costs of biopharmaceuticals are a massive problem for social health systems, particularly those in Western countries in demographic transition [23]. One approach to reduce the prices of biopharmaceuticals is to produce smaller and less complex molecules by using cheaper production systems, e.g., yeast or bacteria instead of mammalian cells [24,25]. However, bacteria are not optimal for industrial production scales, as bacteria-derived endotoxins carry a high risk of immune reactions [26].

### 2.1. Limitation to the Intravascular Compartment

Another drawback is the limited pharmacokinetic distribution of most mAbs to the plasma (intravascular) compartment. As large molecules, they barely penetrate into tissues, and over 99% remain in the blood plasma [27,28,29]. In the absence of an active transport system, molecules diffuse via tissue barriers depending on their hydrodynamic radius; hence, smaller peptides and proteins penetrate into tissues much more efficiently [30,31]. This is of particular interest for cancer therapy; mAbs can rather effectively attack metastatic cells circulating in the bloodstream but can hardly penetrate and thereby reduce solid tumors. Therefore, most mAbs are used as add-on therapies against cancer. Smaller entities with higher tumor penetration may be used as diagnostic biomarkers and as more efficient drugs. Finally, many mAbs are currently evaluated for targeted drug delivery despite their size and costs.

### 2.2. Limited Tissue Penetration

Further, the missing or minimal ability of these proteins to cross cellular barriers without a carrier restricts their therapeutic use on well-exposed and accessible targets, as they cannot cross, for example, the blood–brain barrier (BBB) in sufficient amounts [32]. As a consequence, only roughly 0.1–0.4% of an individual IgG plasma concentration can be found in the CNS [33,34,35], and this can only be enhanced by using either trans-cellular transport mechanisms like transferrin- [36] or insulin receptor-mediated transcytosis [37], or by exploiting pharmaceutical particulate formulations [38,39]. On the contrary, antibody formats with a reduced size, such as Fab, single chain variable fragments (scFvs) or sdAbs can more efficiently diffuse into tumors and cross specific tissue barriers, e.g., the BBB, without losing their main properties, such as affinity and specificity for their antigen [30,40,41]. Since these formats all bind their antigens via variable domains it seems reason able to reduce the size of full-length IgGs by removing the constant domains (Figure 1). 

## 3. How to Overcome Biodistribution Limitations of Full-Length IgGs 

Of late, there has been an increasing number of approaches to tackling the above-mentioned drug delivery challenges, not only with different drug delivery systems but also with so-called scaffolds, i.e., proteins that display a scaffold-like structure, such as antibodies and Ig domains [42,43,44,45]. Brought together, the aspects of a good biodistribution can be manifold, and only some of them are described with regard to antibodies. 

### 3.1. ScFv 

Attempts to create stable and minimally sized antibody-derived fragments led to the construction of scFv fragments with a pair of VHs and VLs connected by a flexible 15–20 amino acid polypeptide linker [46,47] (Figure 1b). For example, the scFv Brolucizumab, directed against VEGF, is approved for the treatment of macular degeneration, and DLX105, directed against tumor necrosis factor alpha, demonstrated a positive outcome on psoriasis biomarkers in a clinical study [48,49]. Polymers can be obtained from scFvs. Diabodies, formed by linking two scFvs together, are able to bind to two antigens with a higher avidity than monomeric scFv antibodies. Diabodies can be either bivalent or bispecific, meaning that each scFv can bind to the same antigen or has a different antigen target, respectively [47]. The tandem scFv Blinatumomab, directed against CD19 and CD3, is approved as a bispecific T cell engager for acute lymphoblastic leukemia [50]. The approval of a third scFv, Oportuzumab monatox, is currently pending. Oportuzumab is directed against EpCam and fused with *Pseudomonas aeruginosa* exotoxin A for the treatment of a specific bladder cancer [51]. L19IL2 is a fully human fusion protein, consisting of the scFv of the L19 antibody specific to the alternatively spliced extra-domain B of fibronectin, fused to human interleukin-2. This scFv fusion protein is being developed for the treatment of advanced metastatic melanoma [52]. It should be noted that scFvs are very often used as part of the chimeric receptors of CAR (chimeric antigen receptor) T or NK cells [53].

### 3.2. Single-Domain Antibodies

#### 3.2.1. sdAbs from Camelids

In 1993, Hamers and his colleagues identified an unusual immunoglobulin in camel serum which lacked the light chain [54]: heavy chain-only antibodies (HCAbs). These HCAbs are found in *Camelidae* (*Camelus dromedarius*, *Camelus bactrianus*, *Lama glama*, *Lama vicugna*, *Lama guanaco*, *Lama alpaca*) [47]. In addition to the lack of LC, the first constant domain of the heavy chain (CH1) is also absent in HCAb (Figure 1c). The variable domain of this antibody, known as VHH (VH of HCAb) or nanobody, is roughly 15 kDa in size and is responsible for binding to antigens (Figure 1d) [55,56]. Since then, VHHs have gained the attention of researchers as they have remarkable unique properties compared to other antibodies [57]. The conserved hydrophobic amino acids in VHs, which interact with VLs are substituted by hydrophilic amino acids in VHHs. Up to now, many high-affinity VHHs against a wide variety of antigens have been produced from different types of antibody libraries [58] with a high protein stability [59]. Such libraries can be obtained from naïve or immunized animals or designed synthetically [60].

In early 2019, the US Food and Drug Administration approved Cablivi (caplacizumab), a VHH dimer for the treatment of acquired thrombotic thrombocytopenic purpura, associated with excessive blood clotting in capillaries [61,62,63]. Caplacizumab was the first approved sdAb. Caplacizumab was discovered by Ablynx, which was later bought by Sanofi in January 2018 for €3.8 billion [62]. Recently, a very low risk for immunogenicity was demonstrated in clinical trials with two diagnostic VHHs directed against human epidermal growth factor receptor 2 (HER2) and against the macrophage mannose receptor (MMR) [64].

#### 3.2.2. sdAbs from Sharks

In 1995, a new antibody molecule which possessed desirable protein scaffold characteristics was found in the serum of the nurse shark (*Ginglymostoma cirratum*) by Flajnik and his group and was termed immunoglobulin new antigen receptor (IgNAR, Figure 1e) [65]. Up to now, IgNAR has been found in different shark species, e.g., the small-spotted catshark (*Scyliorhimus canicula*), the bamboo shark (*Chiloscyllium plagiosum*), the spiny dogfish (*Squalus acanthias*) and the wobbegong (*Orectolobus maculatus*) [66,67,68,69,70].

IgNAR is a homodimeric heavy chain-only complex, which also naturally lacks the LC, containing one variable (VNAR) and five constant (CNAR) domains. These homodimers are held together by C_1_NAR and C_5_NAR domains [65,71]. The variable domain of this antibody, the VNAR, has a characteristically small size of only 12 kDa (Figure 1f) [72], making it the smallest known natural domain with full antigen-binding capacity [70]. VNARs display sequence homology with vertebrate Ig domains, primarily T cell receptors (TCR Vα) and Ig Vκ (light chain kappa variable region), and share structural similarities with TCR Vα, Vλ (light chain lambda variable region) and VH [73]. The VNAR domain with β-sandwich folding consists of four (hyper)variable loops, called CDR1, hypervariable loop 2 (HV2), HV4 and CDR3. Due to the deletion of a large part of the FR2 region and a part of CDR2 by somatic mutations, the CDR2 domain in VNARs is shorter than the common CDR2 in other vertebrate antibodies. Moreover, as a result of this deletion, variable domains in IgNARs have only eight instead of the 10 β-strands that are commonly found in the Ig superfamily, the missing CDR replaced by very short HV2 and HV4 strands [74,75,76]. 

Higher diversity in the composition and number of amino acid residues are found in CDR3, and this loop is the main part responsible for the antigen-binding process [73]. Based on the maturation of the shark’s immune system and the position of non-canonical cysteine (Cys) residues, four different types (I-IV) of IgNARs have been identified [77,78]. The classical canonical disulfide bond between FR1 and FR3, which is found in common variable domains, is also found in all types of VNARs [75].

- Type I has two or four Cys residues in CDR3 and two Cys residues in FR2 and FR4. This results in the formation of disulfide bonds between FR2-CDR3 and CDR3-FR4. In addition to increasing the stability of this type of domain VNAR, it is assumed that the extra disulfide bonds also stabilize the structure of the loop–antigen complex [79].
- In Type II (or Type IIa), in addition to a canonical disulfide bond, there is a single Cys residue in CDR3 and also a Cys in CDR1 [80]. These Cys residues form an extra disulfide bond within the molecule, which brings CDR1 and CDR3 loops close to each other, specific to this type of VNAR.
- Type III is the most dominating antibody type in neonatal sharks but is rarely found in adult sharks [81,82]. Type III VNARs have the same disulfide bonds as Type II. Contrary to a Type II VNAR, however, they have less diverse CDR3s and a conserved tryptophan residue in the vicinity of Cys in CDR1 [79].
- Type IV (or Type IIb) VNAR hold only the canonical conserved Cys in their structure. Thus, the topology structure of this antibody domain is more flexible compared to other types of VNAR, except type III. Moreover, another type of IV VNAR which lacks the non-canonical cysteine residues but has an invariant tryptophan residue in CDR1 has been identified. This type of VNAR is referred to as Type IIIb [83,84].

High-affinity binders have been selected from all other types of VNARs [85,86,87,88].

Companies such as Elasmogen Ltd., AdAlta and Pfizer are actively pursuing shark antibody research and applications [62,89]. A humanized version of VNAR called SoloMERs^TM^ was introduced by Elasmogen and is said to bind to intracellular targets [90]. Like all sdAbs, these humanized VNARs are highly stable and are able to bind to their target antigens even after exposure to high temperatures and extreme pH levels. More interestingly, they possess the ability to penetrate into the nucleus of a cell. However, these features are not unique to VNARs but are advantages shared by all sdAbs.

The Australian company AdAlta call their humanized VNARs ‘i-bodies’, and they are not only focusing on CNS delivery of VNARs but also other unmet medical needs. AD-114 is the most advanced i-body and the leading candidate directed against CXCR4 for the treatment of fibrosis [91,92]. In collaboration with the Dutch biotech company Crossbeta Biosciences, anti-amyloid beta shark i-bodies have been licensed and developed by AdAlta. 

### 3.3. Human-Derived sdAbs

Despite the benefits of the above-mentioned sdAbs over HCAbs, these formats are derived from xenogeneic sources, and it is of vital importance for therapy in humans to decrease the immunogenic potential by humanizing the sdAbs. Alternatively, human sdAbs can be achieved by expressing isolated human VH or VL domains. Davies and Riechmann were the first group who developed human VH libraries [96]. To avoid the problem regarding the solubility of these antibody libraries, they ‘camelized’ the antibody gene fragments by substitution of the hydrophobic amino acids in the framework-2 (FR2) region with hydrophilic amino acids of VHHs available in that region. Another attempt in the construction of VH libraries was made by Reiter in 1999 [97]. This library was produced from a VH mouse with natural mutations that made the domain more soluble. Later, this approach was used by Domantis Ltd. (now part of Glaxo Smith Kline) to synthesize a vast human VH library [98]. Barthelemy and his group employed phage display technology to establish optimal mutations to generate a soluble, stable, well expressed human VH library [99]. The library contained randomized amino acid sequences at hotspot positions in FR2 and CDR3 of human VH-4D5 which were different from natural mutations found in VHHs. In addition, human VL domains were also used as protein scaffolds to construct synthetic antibody libraries [100]. 

Recently, a second intradomain disulfide linkage occurring in camelid VHHs was introduced in human VL sdAbs and resulted in a higher thermostability of VL proteins [101]. In addition, human-derived sdAb libraries were shown to provide clones with good manufacturability [102].

## 4. General Considerations on Therapy with Antibodies and Their Derivatives

### 4.1. Molecular Size and Size of the Paratope—Size Does Matter

It is well accepted that the hydrodynamic diameter of therapeutic agents correlates with their tissue distribution: the larger the diameter, the lower the permeation [33,103]. Moreover, size-dependent uptake of nanoparticles has been investigated in different cell types [104,105]. Therefore, sdAbs may have potential as tissue-penetrating antibodies [75] due to their small size and excellent physicochemical properties [43,106]. We have unpublished data that VNARs demonstrate significantly higher permeation rates than full-size IgGs in a transwell permeation experiment across a confluent layer of nasal RPMI 2650 cells [105,106].

While the impact of molecular size has been well evaluated for small molecules, hardly any critical reports exist on its effect on biological activity when sizing the paratope. The human antigen-binding regions in antibodies consist of one VL and one VH domain within a total of six CDRs (see Figure 1a,b). A VNAR has only two CDRs, although other regions may also take part in antigen binding (Figure 1f). Despite the lower molecular mass of nanobodies, the mean total surface area of their paratope is roughly 92% of the surface area of an IgG antigen binding site [107]. However, the spatial orientation of the paratope is of high relevance to achieving the desired affinity and thereby bioactivity. In classical IgGs, the CDRs of VH and VL create a surface with 600–900 Å^2^, which can form a cavity, a groove or a flat surface. These paratopes can recognize small molecules, linear peptides and larger antigens, respectively [108]. To compensate for the absence of the VL domain, nanobodies and VNARs have larger loops, especially CDR3 [109]. One of the main characteristics of the larger and more flexible CDR3 of sdAbs is that it allows the recognition of unique conformational epitopes, including active sites of enzymes, cavities of receptors or unfolded proteins [59,109,110]. This flexible loop may bind to the antigen with similar affinities, for instance, IgG [111]. Some records of VNARs with affinities in a low nM range have been reported from primary antibody repertoires without in vitro affinity maturation [73,74,112]. However, the highest affinity in the pM range was obtained from the immunized library against albumin [87]. When combining biparatopic sdAb, K_D_ levels in the pM range are achieved more easily [113]. The paratopes of conventional antibodies directed against folded proteins are mostly flat or concave [114], while sdAb paratopes can adopt both flat and convex topologies [115,116]. However, the binding affinity is predominantly dependent on the size of the interface area between paratope and epitope, and the footprints of sdAbs on antigens are smaller than those of conventional antibodies given that their paratopes are roughly half the size [117]. Hence, this is a clear limitation of monomeric sdAbs when it comes to clinical efficacy.

Of course, multimerization of sdAbs can increase their binding properties via avidity effects; however, multimerization increases their molecular size. Recently, new formats of anti-TNFα VNARs have been isolated and characterized by magnitude improvements in affinity compared to Adalimumab [118]. The parental monomers of these VNARs were derived from an immunized nurse shark which is able to neutralize TNFα in the cell-based assay at nM concentration. Bivalent-bispecific formats of these VNARs were constructed in two formats: bispecific VNARs were fused to the N-terminus of the human IgG1 Fc domain (Quad-Y™) or fused to both ends of the Fc domain (Quad-X™) with a molecular weight of less than 100 kDa when expressed in mammalian cells. They were capable of neutralization of cytokine-mediated cytotoxicity at concentrations of 2–5 pM, ten times better than Adalimumab.

Despite tissue penetration, the lack of the Fc domain enables targeting molecules to avoid the risk of Fc receptor-mediated uptake; to deliver toxins or other drugs to (tumor) cells, antibody drug conjugates (ADCs) are used. Here, also, a smaller size could improve the bioactivity of ADCs. Fang et al. created a VHH–drug conjugate to target MHC II complexes on the murine A20 lymphoma cell line. The coupled Mertansine, a thio-containing Maytansine derivative, showed better IC_50_ values on A20 cells (36 nM) compared to cell lines lacking MHC II proteins (500 nM). In contrast, uncoupled Mertansine showed similar cytotoxicity values for all cell lines. Hence, the VHH is responsible for a more selective degradation of cells, which makes it efficient in B cell lymphoma therapy. A similar approach is possible for any antigen that distinguishes a tumor and a normal cell. There are also conjugates against myeloma and leukemia cell lines [119,120]. Strikingly, sdAb–drug conjugates are believed to have an improved safety profile, since unspecific uptake via Fc receptors will not occur. Currently, companies such as Antikor and Australia SME Avipep, who explore diabody–drug conjugates, are actively working on developing scFv-based drug conjugates [22]. 

### 4.2. Elimination and Half-Life Are also Important Criteria for Drug Delivery

Although the small size is beneficial for tissue penetration, it hides a disadvantage that also affects drug delivery for chronic diseases: the smaller the molecule, the easier and faster it is removed from the body by renal clearance and lysosomal degradation. The kidney serves as a molecular filter with a cut-off of 40–60 kDa depending on the hydrodynamic radius, and smaller proteins are removed in urine [121]. In contrast, full-length IgGs persist longer in the bloodstream [44]. Here it has to be noted that there is another aspect that must be considered regarding the pharmacokinetics of an IgG: due to the Fc fragment that a full-length IgG harbors in contrast to a nanobody, it is recycled by neonatal Fc receptors (FcRn). After internalization, the FcRn binds the IgG at a slightly acidic pH inside a cell and releases it when the pH is increased to neutral levels on the cell surface. Thereby, full-length IgGs escape from lysosomal degradation and display a serum half-life of up to three weeks [122]. 

It is known that the clearance rates of smaller molecular and antibody fragments are higher than those of full-length antibodies. The serum half-lives of these molecules differ significantly: the highest plasma half-life is reported for full-length IgGs with 110 h, 5 h for mini body (80 KDa), 1 h for an scFv (27 KDa) but only 3–9 min for a VH or VL domain (13 KDa) [121,123,124]. Therefore, it is a challenge to use a monovalent sdAb without any modifications in treatment of chronic diseases.

However, it is possible to increase the half-life of sdAb by increasing the hydrodynamic size above the kidney threshold through chemical modification by methods including PEGylation [125], Pro, Ala, Ser (PAS)ylation [126], glycosylation [127], hydroxyethyl starch (HES)ylation [128] or by using FcRn-mediated recycling through the generation of Fc fusion proteins, albumin fusion proteins or fusion to albumin binders [87,129]. 

Moreover, continuous drug supply and controlled continuous release from particles or from a depot are options to avoid increasing the hydrodynamic diameter of sdAbs. The challenge here is that the release from the depot has to be as fast as the terminal elimination kinetics to achieve steady-state drug levels. 

Some organs or tissues have unique elimination conditions and thereby the terminal half-lives of drugs in these organs can be different from plasma half-lives. The eye, for instance, is such an organ, and the distribution of intravitreal drugs in the vitreous humour is conditioned by its intrinsic characteristics, such as volume and composition, as well as by the properties of the drug (charge, molecular weight and protein binding capacity) [130]. The elimination of drugs from the vitreous humour can occur via two different routes, either by metabolism or by disposal into the systemic circulation. The scFv Brolucizumab has an intravitreal half-life of roughly 3 days and hence can be used in a clinical setting without half-life extension [131].

Finally, the multimerization of an sdAb with its target can also increase its half-life; the approved VHH dimer Caplacizumab binds to van Willebrandt factor (vWF) in both its active and inactive forms, thereby blocking the interaction of vWF with platelets but also increasing the total hydrodynamic diameter and hence the half-life [61]. 

### 4.3. Higher Stability Enables Aerosolization and Airway Delivery

While full-length IgGs can aggregate during aerosolization, scFvs and sdAbs are considered to be more stable against mechanical shear stress due to their compact size [30,132,133]. This is of particular importance for intranasal delivery to the brain with protein aerosols. Therefore, a summary of studies on aerosolization with antibodies and their derivatives is presented here.

SdAbs have been developed to fight infectious diseases, such as severe acute respiratory syndrome coronavirus 2 (SARS-CoV-2) and influenza [134]. ‘AeroNabs’, aerosol-formulated nanobodies, could be used with a nasal spray or inhaler for personal protection against coronavirus disease 2019 (COVID-19) [135]. These VHHs were selected from a yeast surface-displayed library of synthetic nanobodies which are able to disrupt the interaction of SARS-CoV-2 spike-to-host cell receptor. The affinity of selected sdAbs was increased by producing a highly robust trivalent VHH molecule, mNb6-tri, with the ability of binding to the spike protein with femtomolar affinity and neutralization of infection in the picomolar range, with remained function and high stability after aerosolization, lyophilization and heat treatment. Koeing et al. obtained four VHHs (E, U, V and W) from an alpaca and a llama immunized against the spike protein of the SARS-CoV-2 using phage display [136]. The crystal structure of an antibody–spike protein complex revealed that the VHHs are able to target two distinct epitopes on the receptor binding domains of the spike protein. The selected VHHs were able to bind the viral target protein with an equilibrium dissociation constant between 2 and 22 nmol and could prevent SARS-CoV-2 infection by 50% in a plaque-reduction assay at concentrations ranging from 48 to 185 nmol [136,137]. Three out of the four nanobodies were also able to block SARS-CoV-2 from engaging angiotensin converting enzyme 2 on host cells, owing to the position of the epitopes to which they bind and their mechanism of interaction with the receptor-binding domain. According to the authors, the multivalent nanobodies demonstrate thereby a neutralizing activity 100 times higher than monovalent nanobodies.

Nevertheless, the coronavirus pandemic has also accelerated the development and clinical trials of full-length IgGs for inhalative or intranasal airway delivery [138,139].

## 5. CNS Drug Delivery with Full-Length IgG, scFv and sdAb

The delivery of drugs which can reach the CNS in sufficient concentrations is still a significant challenge in the treatment of CNS diseases [140]. One of the most selective barriers surrounding the CNS is the BBB formed by endothelial cells with tight junctions (TJ) and adherence junctions (AJ). The BBB does not only exclude large molecules; the FcRn expressed in the BBB and the blood–cerebrospinal fluid (CSF) barrier also contributes to the fast elimination of full-length IgGs from the brain [141]. Excluding immunological proteins, such as antibodies, from the brain is an important function of the BBB, preventing proinflammatory signs, e.g., swelling. Since the brain is surrounded by bony structures, swelling can lead to severe tissue damage. Therefore, the CNS is also referred to as an immune-privileged zone. Hence, smaller proteins, such as nanobodies and antibody derivatives without an Fc domain, should have a better potential for neurological and psychiatric indications [142].

Several drug delivery strategies have been introduced to cross the BBB: a paracellular route or passive diffusion by disruption of the BBB, a transcellular route by enhancing transcytosis using molecular BBB shuttles or direct delivery bypassing the BBB with intrathecal, intraventricular or intranasal delivery [143,144,145]. While nanobodies have been often discussed in relation to crossing the BBB, the number of experimental validations is rather low. However, the results of one study indicated that, following intravenous injection, two VHHs targeting extracellular amyloid deposits and intracellular tau neurofibrils bypassed the BBB in transgenic mouse models [142]. This is a promising achievement which could make the early-stage diagnosis of brain-related diseases, such as Alzheimer’s disease, possible. Furthermore, a VHH fusion protein with green fluorescent protein was designed to target glial fibrillary acidic protein, a protein expressed on astrocytes. This VHH was able to cross the BBB in vivo and reached the astrocytes after intracarotid and intravenous injections [146]. The authors discussed the possibility that the basic isoelectric point of a VHH could play a role. More recently, in a study conducted by Miyashita et al., a VHH was delivered into cultured neurons to target and neutralize botulinum neurotoxins. Interestingly, when this nanobody was applied in mouse models with lethal doses of neurotoxins, the duration of muscle paralysis decreased and the mice were rescued within hours from systemic toxicity by restoring the function of the muscles [147]. Nevertheless, the transport mechanisms across the BBB or into the neurons have not been elucidated in all of the above-mentioned studies.

The active transport of molecules, such as proteins and peptides, through the BBB is mediated by three mechanisms: adsorptive-mediated transcytosis (AMT), transporter-mediated transcytosis, and receptor-mediated endocytosis (RMT) [148]. The human insulin receptor is known to shuttle insulin over the BBB by transcytosis. In addition, IgG antibodies binding this receptor have been shown to be shuttled simultaneously to the CNS [149]. Several studies have shown so far that mAbs targeting insulin receptors are effective in animals to transport a model drug across the BBB [37,150,151]. Engineering of receptor-binding molecules and reduction of size and complexity is believed to improve transcytosis and thereby brain delivery [150,152,153]. Additionally, several sdAbs were shown to be transported into the CNS via this BBB shuttle; for instance, a patented camelid IGF1R (insulin-like growth factor 1 receptor)-specific VHH was able to transmigrate across the BBB by RMT in in vivo and in vitro models [154]. In a study conducted by Muruganandam and his group, a naïve llama library was used to pan against human cerebromicrovascular endothelial cells (HCEC) [155]. Two selected VHHs, FC5 and FC44, were shown to selectively bind HCEC and transmigrate across them via the RMT mechanism and were able to reach the brain in mouse models after IV injection. The transport of FC5 across human brain endothelial cells was polarized, charge independent and temperature dependent. FC5 was taken up by HCEC via clathrin but not caveolin-1 mediated pathways [156]. Finally, Lundbeck in collaboration with Ossianix established the VNAR shuttle TXP1 against the transferrin receptor (TfR1), which can be delivered to the brain by bypassing the BBB via RTM [157].

Receptor-mediated uptake and transcytosis can be combined with carrier-mediated targeted drug delivery. Highly specific scFv or sdAb against receptors at the BBB, such as the insulin or low-density lipoprotein (LDL) receptors, are loaded into liposomes or other nano/microparticles to target and guide an encapsulated drug cargo into the specific tissue. Therefore, sdAbs binding to receptors at the BBB and inducing transcytosis are an interesting option for CNS delivery. VCAMelid against mouse vascular cell adhesion molecule 1 (VCAM-1) was used as a carrier to deliver therapeutic protein superoxide dismutase (SOD-1) and a nanocarrier (liposome) into the brain of mouse models in the format of both monovalent and bivalent nanobodies. This delivery was higher when a VHH was used as a targeting receptor [158].

The Rotman group used modified liposomes to deliver VHHs to the brain. In their study, two different formulations of glutathione targeting PEGylated liposomes were used to deliver an anti-amyloid VHH as cargo. The encapsulated VHHs were intravenously injected into a murine Alzheimer’s disease model and the presence of VHHs quantified in the CNS. While from 0.001% of the injected dose was found for an unformulated VHH without carrier, the encapsulation in liposome achieved significantly higher levels, from 0.015% up to 0.094% [159]. These results indicate that liposomes with specific formulations could be useful carriers/shuttles for administrating large therapeutic drugs over the BBB into the brain.

### 5.1. Intranasal Nose to Brain Delivery

**Figure 2:**
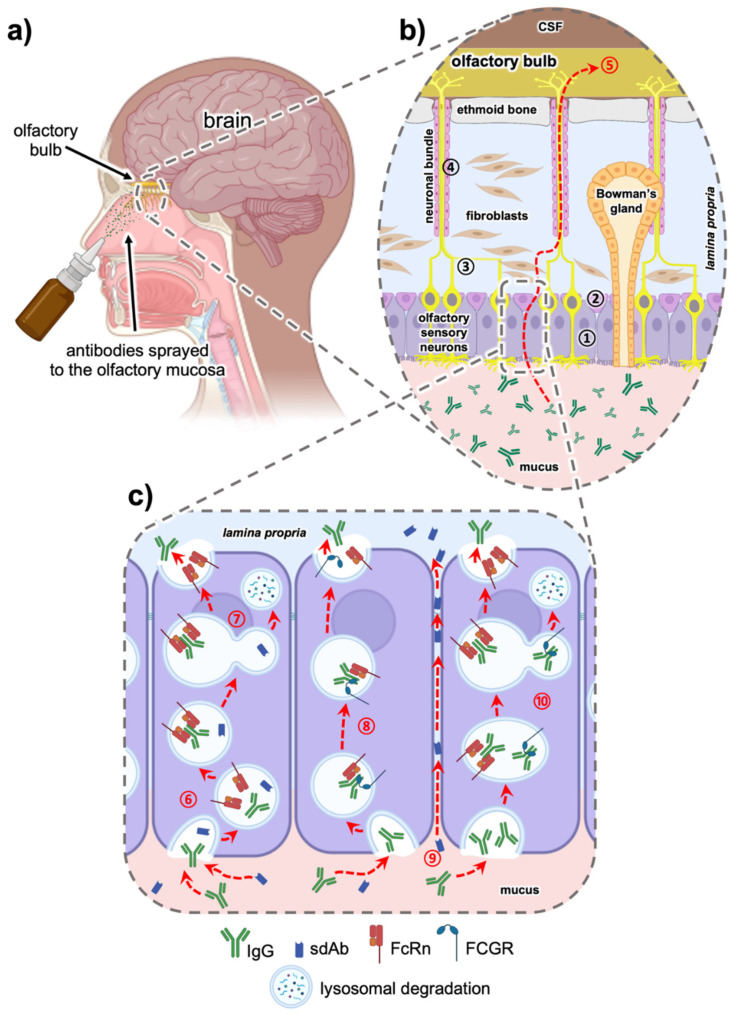
Intranasal delivery via the olfactory region of antibodies and their derivatives. Anatomical overview of the human nasal cavity, with olfactory nerve endings within the olfactory mucosa projecting to the olfactory bulb (**a**). A magnification of the olfactory mucosa is shown in (**b**). The epithelial layer consists of sustentacular ① and basal cells ②. The hallmarks of this epithelium are the olfactory sensory neurons and Bowman’s glands. The axons ③ of the olfactory sensory neurons are collected in the neuronal bundles projecting to the olfactory bulb. These bundles are surrounded from olfactory ensheating cells ④ and transverse the ethmoid bone into the cranial cavity. When drugs such as antibodies are administered to this olfactory region, the proteins can be transported from the nasal mucosal surface to the brain (nose-to-brain) as demonstrated with the red dotted arrow ⑤. The state-of-the-art hypothesis concerning the olfactory transport of full-length IgG antibodies [160,161] or sdAbs (unpublished data) [105] is shown in (**c**): uptake via endocytosis and binding of IgG to neonatal Fc receptors (FcRn) via their Fc domain under acidic conditions ⑥. An SdAb devoid of Fc cannot be bound and is therefore sorted into the lysosomal pathway ⑦. The endosomes containing FcRn-bound IgG transmigrate the polar sustentacular cell where they fuse with the basolateral plasma membrane. At physiological pH, the IgG is released from the FcRn diffuses to the lamina propria. In addition, a transcytosis pathway based on a mixed co-transport of full-length IgG via FcRn and Fc-gamma receptors (FCGR) could be possible ⑧. Due to their smaller size, sdAbs can enter paracellular pathways between the epithelial cells ⑨ to reach the lamina propria. Finally, IgG bound to FCGR only can be sorted to the lysosomal pathway and degraded ⑩. Image created with BioRender.com.

CNS drug delivery via the olfactory, trigeminal or optic nerves bypasses the BBB and could thereby dramatically advance the CNS drug delivery. In particular, intranasal nose to brain (N2B) delivery has emerged as an exciting and attractive minimally invasive delivery option (Figure 2a). As we have recently shown, after region-specific deposition at the olfactory region, full-length IgGs can use either intracellular or paracellular pathways to cross the epithelial layer (Figure 2b). Intracellular pathways across the olfactory epithelium include the endocytosis pathway into epithelial cells and/or olfactory neurons and transcytosis to the lamina propria [160,161]. In the extracellular pathway, molecules use paracellular diffusion through either the olfactory or respiratory tight epithelial junctions to reach the basal lamina [162]. From here, the IgGs are observed along neuronal bundles that project to the olfactory bulb.

It is well accepted that lipophilic molecules are predominantly transported along transcellular pathways, while more polar molecules appear to have a higher preference for the paracellular pathway [163,164]. However, both pathways are dependent on diffusion, which is the limiting factor when it comes to large molecules, such as antibodies. The impact of molecular size on the probability of crossing an epithelial barrier in the absence of other transport mechanisms is well known [165]. Up to now, intranasal delivery of small proteins, such as insulin [166], scFvs [167] and nanobodies [168], was demonstrated. Benedict et al. showed that administered insulin had a positive effect on mood and also memory in patients with mild cognitive impairment and Alzheimer’s disease [169]. Intranasally administered insulin exhibited positive clinical effects on memory [170] and hypothalamic functional connectivity [171]. They found that the intranasal insulin did not enter the blood and did not affect the plasma glucose levels. It can be concluded that intranasal administration decreases the possibility of side effects of systemic application.

Only a few other reports used larger proteins like IgGs and found a rapid distribution to the olfactory bulb and other brain areas, implicating olfactory and trigeminal pathways [172,173,174]. Unfortunately, most intranasal in vivo studies lack pharmacokinetic data, and even if quantitative data are shown, they do not disclose intranasal bioavailability [175]. Therefore, the effects of molecular size and physicochemical properties, e.g., polarity, and active or passive transport mechanisms have hardly been investigated in the context of intranasal delivery. To predict the efficiency of intranasally delivered biopharmaceuticals, it is crucial to estimate the percentage of the dose that is able to cross the nasal mucosa in relation to the bioactivity of the biopharmaceutical.

An scFv against tumor necrosis factor α (TNFα) was previously delivered to the brain via an intranasal route and some pharmacokinetic parameters were determined for different brain regions. TNFα is a well-established and well-studied target with a high clinical impact and application. TNFα is a critical inflammatory cytokine that plays a pathological role in acute and chronic diseases of peripheral organs, such as rheumatoid arthritis and Crohn’s disease, as well as diseases of the CNS, such as Parkinson’s disease, Alzheimer’s disease and multiple sclerosis [176]. The results of these experiments showed that the maximum concentration reaching the brain was 1.1 to 1.2 μg/mg of the total applied scFv (400 μg) [167].

Gomes et al. successfully delivered an anti-transthyretin single-domain antibody intranasally in mice [168]. After delivery of 400 µg of this nanobody in transthyretin knock-out mice, it distributed to all brain areas, but the highest levels were observed in the olfactory bulb and the central parts of the brain. Interestingly, this nanobody was even detected in the spinal cord after intranasal delivery. Moreover, they found that the highest concentration of this antibody reached the brain and cerebrospinal fluid within one to two hours. A comparable pattern of this distribution was shown for transthyretin in wild-type mice.

### 5.2. The Role of the Fc Domain in Intranasal Transmucosal Delivery—Is it a Friend or a Foe?

Fc-gamma receptors (FCGR) belong to the immunoglobulin superfamily. It is well known that the Fc domain of an IgG molecule binds to FCGR and relevant complement factors, thereby triggering immune effector functions, such as antibody-dependent cellular cytotoxicity (ADCC) and complement-dependent cytotoxicity (CDC). Moreover, Fc-related transport molecules, e.g., FcRn, have been well characterized. Since the nasal mucosa is highly exposed to microbes and viruses, several immune cells armed with different FCGR are resident in this mucosa. Recently, our group has demonstrated that the use of ex vivo mucosa and primary cells arising from this tissue are valuable models for studying the uptake and transport pathway for intranasal delivery [160,177]. The nasal mucosa harbors FcRn and different FCGRs in epithelial, basal and immune cells, but also olfactory ensheathing cells [39,161], facilitating the transport of IgG from the apical to the basolateral side in polarized cells [160,178]. As shown in Figure 2c, FcRn-mediated transcytosis in an acidic environment increases the uptake of IgG from the mucosal surface [160,179]. On the other hand, we were recently able to demonstrate that FCGR2 directs IgGs to the lysosomal pathway, such that they are degraded rather than being distributed to the CNS [161].

Currently, it is not clear whether the interaction with Fc receptors is a friend or a foe for intranasal drug delivery, since the interaction of IgG molecules with immune cells is very likely. We have observed that mucosal lymphoid follicles are spared from full-length IgG-immunoreactivity [160]. The suspected underlying mechanism is uptake of the IgGs via FCGR, lysosomal degradation and the presentation of eventually captured antigens via MHC. Though this is a highly important process for mucosal immune surveillance, it is a potential source of adverse effects and immunogenicity for N2B CNS delivery with full-length IgGs. Fc fusion molecules would also become eligible for FcRn recycling [180].

Hence, a potential mitigation strategy could be to use either IgGs with mutations that abolish Fc receptor interaction or to use antibody formats without Fc domains, such as sdAbs. However, it is rather likely that without the transport via the FcRn receptor system the penetration of the mucosa will be rather low, despite the advantage of their smaller size.

## 6. Conclusions and Outlook

The effect of the size of antibodies and their derivatives on permeability and tissue penetration is unquestionable, and hydrodynamic diameter is the main reason for the absence of transport mechanisms. Smaller antibody formats have essential characteristics for CNS delivery which can be developed into novel carriers to facilitate drug transport across the BBB. Although sdAbs and other small formats are clearly superior in trans-mucosal delivery under in vitro conditions, pharmacokinetic factors, such as half-life, are important for efficacy in vivo. Based on recent results of in vitro, ex vivo and in vivo studies, the passive diffusion of sdAbs might be countervailed by transport-related processes via the Fc domain involving full-length IgGs in the airway mucosa. The Fc domain interacts with different Fc receptors, such as FcRn or members of the FCGR family. These receptors mediate transcytosis, rescuing full-length IgGs from the endosomal system but also leading to their lysosomal degradation. Nevertheless, sdAbs, being less complex and more stable during aerosolization, have significant potential for inhalative drug delivery, and could be made competitive with reduced manufacturing costs. In conclusion, exploring the potential and limitations of sdAbs for CNS drug delivery is highly relevant in the context of developing treatments for diseases such as brain tumors or neurodegenerative disorders. 
